# Ethnic specific association of the *CAV1/CAV2* locus with primary open-angle glaucoma

**DOI:** 10.1038/srep27837

**Published:** 2016-06-14

**Authors:** Shi Song Rong, Li Jia Chen, Christopher K. S. Leung, Kenji Matsushita, Liyun Jia, Atsuya Miki, Sylvia W. Y. Chiang, Pancy O. S. Tam, Noriyasu Hashida, Alvin L. Young, Motokazu Tsujikawa, Mingzhi Zhang, Ningli Wang, Kohji Nishida, Chi Pui Pang

**Affiliations:** 1 Department of Ophthalmology and Visual Sciences, the Chinese University of Hong Kong, Hong Kong, China; 2 Department of Ophthalmology and Visual Sciences, Prince of Wales Hospital, Hong Kong, China; 3 Department of Ophthalmology, Osaka University Graduate School of Medicine, Suita, Osaka, Japan; 4 Beijing Tongren Eye Center, Beijing Tongren Hospital, Capital Medical University, Beijing, China; 5 Joint Shantou International Eye Center of Shantou University and the Chinese University of Hong Kong, Shantou, China

## Abstract

A single-nucleotide polymorphism (SNP) rs4236601 at the *CAV1/CAV2* locus is associated with primary open-angle glaucoma (POAG). Rs4236601 is common in Caucasians but rare in East Asians. Here we conducted a haplotype-tagging SNP analysis followed by replication in a total of 848 POAG cases and 1574 controls drawn from 3 cities in China and 1 city in Japan. Two SNPs, rs4236601 (odds ratio [OR] = 6.25; P = 0.0086) and a tagging-SNP rs3801994 (OR = 1.32; P = 0.042), were associated with POAG in the Hong Kong Chinese cohort after age and gender adjustments. Rs4236601 was associated with POAG also in Shantou (OR = 6.09; P = 0.0037) and Beijing (OR = 3.92; P = 0.030) cohorts after age and gender adjustment, with a pooled-OR of 5.26 (P = 9.0 × 10^−6^) in Chinese; but it is non-polymorphic in the Osaka cohort. SNP rs3801994 showed a similar trend of effect in the Shantou and Beijing cohorts, with a pooled-OR of 1.23 (P = 0.022) and 1.20 (P = 0.063) in Chinese, prior to and after age and gender adjustment, respectively; but it showed a reverse effect in the Osaka cohort (OR = 0.58; P = 0.033) after the adjustments. We have thus confirmed the association of rs4236601 with POAG in different Chinese cohorts. Also, we found a common SNP rs3801994 of diverse associations with POAG between Chinese and Japanese.

Glaucoma is a group of optic neuropathies involving progressive loss of retinal ganglion cells and their axons, resulting in characteristic optic nerve head excavation and visual field defects^1^. It is a leading cause of irreversible blindness worldwide^2^. Primary open-angle glaucoma (POAG) is a major form of glaucoma. POAG has complex etiology, with major risk factors including a high intraocular pressure (IOP), older age, thin central corneal thickness, family history, and genetic susceptibility^3^,^4^,^5^,^6^.

In a genome-wide association study (GWAS), a single-nucleotide polymorphism (SNP) rs4236601 at the *CAV1/CAV2* locus on chromosome 7q31 was identified to be significantly associated with POAG in the Icelandic population and the association was replicated in European decedents and southern Chinese^7^. Notably, the minor allele frequencies (MAFs) and effect sizes of rs4236601 were different across populations. It is a common SNP in Caucasians, with the minor allele A presented in 20–28% of the control subjects, conferring an odds ratio (OR) of 1.1 to 1.3^8^,^9^,^10^. In contrast, rs4236601 is rare in East Asians. For example in Chinese and Korean, the allele A presented in less than 1% of the population, but it conferred a higher OR of 2.9–5.5 for POAG^7^,^11^. Of note, in a Japanese study rs4236601 was found to be non-polymorphic^12^. In addition, the association of *CAV1/CAV2* SNPs, rs4236601^9^,^10^ and rs1052990^9^,^10^,^12^, with POAG was reportedly stronger in female, suggesting a gender-specific association.

In view of the low MAF of SNP rs4236601 in East Asians, there could be other common SNPs at the *CAV1*/*CAV2* locus that are associated with POAG. Therefore, we conducted the present study to investigate the association between common, haplotype-tagging SNPs in this locus and POAG in the Chinese and Japanese populations, and assessed for gender-specific association.

## Results

This study involved a total of 2422 study subjects, including 848 POAG patients and 1574 controls (Table 1). Among them, 890 (454 patients and 436 controls) were southern Chinese recruited from Hong Kong, 854 (123 patients and 731 controls) were southern Chinese from Shantou, 370 (170 patients and 200 controls) were northern Chinese from Beijing, and 308 (101 patients and 207 controls) were Japanese from Osaka. All patients had high-tension POAG and the mean highest IOP was 29.0 ± 8.6 mmHg. The mean IOP of the control subjects was 15.0 ± 2.3 mmHg.

### Association of *CAV1*/*CAV2* with POAG

Two reported SNPs, rs4236601 and rs1052990, and 6 haplotype-tagging SNPs at the *CAV1*/*CAV2* locus were genotyped in the Hong Kong cohort. All SNPs followed Hardy-Weinberg equilibrium (HWE) in the control group (P > 0.05; Supplementary Table 1). SNP rs4236601, which was identified in the GWAS^7^, conferred an increased risk of POAG (A allele, P = 0.0072, OR = 4.72; Table 2), with a population attributable risk (PAR) of 1.47%. A common SNP, rs3801994, showed a borderline association with POAG (A allele, P = 0.036, OR = 1.31; Table 2). This SNP presented in 18% of patients and 15% of controls, conferring a PAR of 4.33%. Logistic regression showed that when conditioned on each other these 2 SNPs were independently associated with POAG: rs4236601 (P = 0.0088, OR = 5.31) and rs3801994 (P = 0.038, OR = 1.22; Supplementary Tables 2 and 3). Moreover, the association of rs4236601 (P = 0.0086, OR = 6.25) and rs3801994 (P = 0.042, OR = 1.32) remained statistically significant after adjusting for age and gender. The other 6 SNPs did not show statistically significant associations with POAG (P > 0.05; Table 2).

Based on the findings in the Hong Kong cohort, we genotyped the SNPs rs4236601 and rs3801994 in the cohorts from Shantou, Beijing and Osaka. We also genotyped the SNP rs1052990 in the Osaka cohort for comparison with a previous study in Japanese^12^ as detailed in the methods. SNP rs4236601 was associated with POAG in the Shantou cohort prior to (P = 0.0079, OR = 4.23) and after adjusting for age and gender (P = 0.0037, OR = 6.09). It had a MAF of 0.49% in controls and 2.0% in patients. Also, rs4236601 showed a significant association with POAG in the Beijing cohort after adjusting for age and gender (P = 0.030, OR = 3.92) and a same trend of effect prior to adjustment (P = 0.057, OR = 2.86; Table 2). The MAF was higher in the Beijing cohort: 1.3% in controls and 3.6% in POAG patients. Of note, rs4236601 was non-polymorphic in our Osaka Japanese cohort. In contrast, rs3801994 was not significantly associated with POAG in the Shantou or Beijing cohort, but their ORs were toward the same direction with that in the Hong Kong cohort (OR = 1.14 in the Shantou cohort and 1.09 in the Beijing cohort; Table 2). Notably, rs3801994 showed an association with POAG in the Osaka cohort after adjusting for age and gender, with an OR toward the opposite direction (P = 0.033, OR = 0.58; Table 2). The MAF of this SNP in the Osaka controls was higher (33%) than that in Chinese (15–19%), and the SNP had a larger PAR 19.29% in the Osaka cohort than in the Hong Kong cohort (4.33%). The common SNP rs1052990^7^,^9^ showed a significant association with POAG after adjusting for age and gender (allele G, P = 0.028, OR = 0.55; Table 2) in the Osaka cohort but not in the Hong Kong cohort, which again highlighted the ethnic difference in genetic associations. Linkage disequilibrium (LD) analysis showed that SNPs rs3801994 and rs1052990 were in weak LD (r^2^ = 0.26). Therefore, the association signals on these two SNPs should be independent.

**Figure 1:**
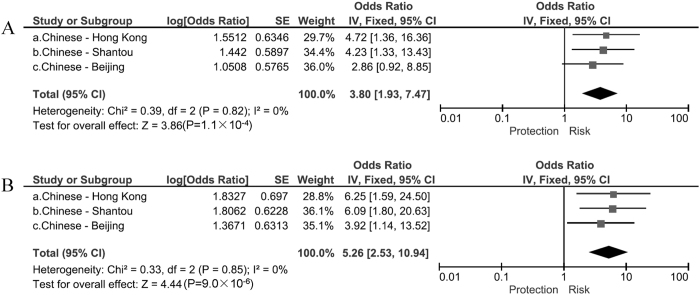
Association of rs4236601 with primary open-angle glaucoma. (**A**) Meta-analysis using unadjusted odds ratios (ORs); (**B**) Meta-analysis using adjusted ORs.

**Figure 2:**
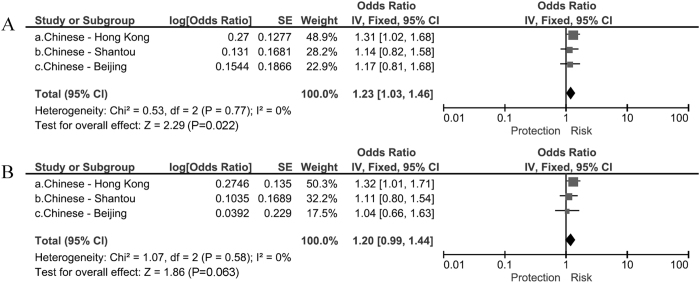
Association of rs3801994 with primary open-angle glaucoma. (**A**) Meta-analysis using unadjusted odds ratios (ORs) (**B**) Meta-analysis using adjusted ORs.

By pooling the data of rs4236601 and rs3801994 from the 3 Chinese cohorts, we found that the SNP rs4236601 was strongly associated with POAG prior to (P = 1.1 × 10^−4^, OR = 3.80) and after adjusting for age and gender (P = 9.0 × 10^−6^, OR = 5.26), with no inter-cohort heterogeneity (I^2^ = 0; Fig. 1). SNP rs3801994 showed a borderline association with POAG (P = 0.022, OR = 1.23, I^2^ = 0) prior to age and gender adjustment, and had the same trend of effect after adjustment (P = 0.063, OR = 1.20, I^2^ = 0; Fig. 2).

### Gender-specific association with POAG

We assessed the association of the *CAV1*/*CAV2* SNPs with POAG in different genders. SNP rs4236601 showed a significant association with POAG in females in the Hong Kong (P = 0.028, OR = 4.94) and Beijing (P = 0.025, OR = 5.76) cohorts, but in males in the Shantou cohort (P = 0.0071, OR = 11.72). The ORs of this SNP were not significantly different between males and females in the 3 cohorts (P > 0.05, Breslow-day test; Table 3). In the pooled samples from the 3 Chinese cohorts, rs4236601 had a significant association with POAG in female (P = 0.0011; OR = 4.84, 95% CI: 1.88–12.45; I^2^ = 0) and a borderline association in male (P = 0.052; OR = 2.83, 95% confidence interval [CI]: 0.99–8.10; I^2^ = 38%; Supplementary Fig. 1). The other 7 SNPs (i.e., rs1052990, rs6466579, rs7801950, rs3779512, rs3807989, rs3801994 and rs1049337) did not show a gender preference in the association with POAG (Table 3).

## Discussion

In this study, we confirmed the association between the SNP rs4236601 at the *CAV1*/*CAV2* locus and POAG in southern Chinese and, for the first time, in northern Chinese. The minor allele A increased the risk of POAG by over 4 fold in southern Chinese and nearly 3 fold in northern Chinese, but it is absent in Japanese. Moreover, we identified a common SNP rs3801994 for POAG, with an OR of 1.23 in Chinese and 0.58 in Japanese and a PAR of 4.33% in Chinese and 19.29% in Japanese. Another SNP, rs1052990 was found to be associated with POAG in the Osaka Japanese cohort (OR = 0.55) but not in the Hong Kong Chinese cohort (OR = 1.33). Thus, our data highlighted the ethnic diversities and independent roles of *CAV1*/*CAV2* SNPs in the genetic susceptibility of POAG.

In Chinese, rs4236601 is an uncommon SNP, with a MAF of 0.43–1.3% in the control populations. But it confers a large risk effect on POAG (OR_pooled_ = 5.26). This is in accordance with the findings in the GWAS^7^ and in a Korean cohort (MAF_control_ = 1.2%; OR = 2.9)^11^. Thus, the risk allele A of rs4236601 has a small PAR in Chinese and Koreans (about 1%), contributing to a small proportion of POAG. In this study, the A allele of rs4236601 was more common in the northern Chinese cohort (MAF_control_ 1.3% in northern Chinese compared with 0.43–0.49% in southern Chinese) and conferred a smaller risk effect (OR = 3.92 in the northern Chinese compared with 6.09–6.25 in southern Chinese). Such intra-ethnic diversity was also noted in other populations. For example, the MAFs and ORs of rs4236601 varied from 20.7% (OR = 1.33) to 28.1% (OR = 1.04) in Caucasian populations recruited from Sweden^7^, the United Kingdom^7^ and the United States^9^. Further studies in more Chinese populations are warranted to determine the allelic distribution and effects of rs4236601 across different geographic regions. Our study also revealed that rs4236601 is monomorphic in the Japanese cohort from Osaka, consistent with the findings in multiple regions of Japan^12^, suggesting that it is not a major gene marker for POAG in the Japanese population. The ethnic differences of rs4236601 in the allele frequency and effect size could be due to natural selection and population drift, which requires further investigation.

Apart from rs4236601, we identified a common, haplotype-tagging SNP, rs3801994, at the *CAV1*/*CAV2* locus that conferred an independent effect on the risk of POAG. In the Hong Kong cohort, it showed a marginally significant association (P = 0.036, OR = 1.31; after adjustment, P = 0.042, OR = 1.32). Though this SNP did not show a statistically significant association with POAG in the Shantou and Beijing cohorts, the ORs were toward the same direction with that in the Hong Kong cohort. The meta-analysis showed that there was no heterogeneity across the 3 cohorts, and the pooled P value was lowered (P = 0.022). Therefore, it is likely that rs3801994 is an associated SNP for POAG. The lack of statistical significance could be due to the limited statistical power in the Shantou (20.3%) and Beijing (16.2%) cohorts, assuming an OR of 1.31 and MAF of 0.15 according to the Hong Kong cohort. In contrast, rs3801994 is more common in Japanese, with a MAF of 0.33 in controls. Intriguingly, it conferred an OR towards the opposite direction in Japanese and had a PAR (19.29%) 4-fold higher than that in the Hong Kong cohort (PAR = 4.33%). This again highlights the ethnic differences in the genetic contribution of the *CAV1*/*CAV2* locus to POAG. The difference in allelic frequencies between Chinese and Japanese found in this study may reflect differential occurrences of glaucoma and clinical features in the two populations, even though both are East Asians. In a previous study in Japanese, a SNP rs1052990 (P = 0.014, OR = 0.71, PAR = 8.79%)^12^ was found to be associated with normal tension glaucoma. Our study suggests that rs3801994 could be another common SNP associated with POAG in Japanese. Notably, however, the association of rs3801994 could not withstand the correction for multiple testing; therefore further studies in both Chinese and Japanese are warranted to confirm its role.

In this study, we have, for the first time, tested the SNP rs1052990 in Chinese POAG. However, in the Hong Kong Chinese cohort, we did not find a significant association. Of note, the minor allele of rs1052990 was G in our Hong Kong and Osaka cohorts, which in accordance with the data HapMap and another study in Japanese^12^. In contrast, allele T was the minor allele in Caucasians^7^,^9^. Moreover, the G allele was protective for normal-tension POAG in the Japanese cohorts in the study of Kato *et al*. (OR = 0.71) and for POAG in the Osaka Japanese cohort in our present study, being opposite to the risk effect observed in Caucasians in previous studies^7^,^9^ and the Hong Kong Chinese in the present study, highlighting the ethnic difference in the effects of rs1052990 on POAG. Of note in this study, a newly identified SNP, rs3801994 also showed a different effect (OR = 0.58) on POAG in the Osaka cohort as compared with the Chinese cohorts (pooled OR = 1.23). The MAF of this SNP in the Osaka controls was higher (33%) than that in Chinese (15–19%), and the SNP had a larger PAR 19.29% in the Osaka cohort than in the Hong Kong cohort (4.33%). Moreover, the SNPs rs3801994 and rs1052990 were in weak LD (r^2^ = 0.26), suggesting the association signals on these two SNPs are independent. Therefore, our study suggests that the effects of SNPs in the *CAV1*/*CAV2* locus are different across different populations, even within East Asians. Further studies in more ethnic cohorts are warranted to confirm this finding.

Among the other 5 SNPs, only 2 SNPs (i.e., rs3779512 and rs3807989) have been reported in independent replication studies^10^,^13^,^14^, while rs6466579, rs7801950, and rs1049337 were tested in POAG for the first time. In this study, SNP rs3779512, which was associated with POAG in Caucasians (OR = 1.15)^10^, did not show a significant association (P = 0.68, OR = 0.92). Also, this SNP was not associated with POAG in an African population^13^, illustrating the ethnic diversity in the genetic contribution of this locus to POAG. Another SNP rs3807989 was not associated with POAG in our study or in African^13^,^14^.

Sex-specific association of rs4236601 has been reported in 2 studies in Caucasians^9^,^10^. These studies reported greater odds ratios and lower P values in females than in males. In our study cohorts, Breslow-day test did not support a significant difference in the ORs between females and males. This might be due to the decreased power after stratification. However, even with limited samples, rs4236601 remained significantly associated with POAG in Hong Kong and Beijing females, and it showed a significant association in the pooled female samples (P = 0.0011, OR = 4.84). In contrast, we found rs4236601 to be associated with POAG in Shantou males (P = 0.0071, OR = 11.72) and have a borderline association in the pooled male subjects (P = 0.052, OR = 2.83). Thus, our data is consistent with the results in the Caucasian studies, in which *CAV1/CAV2* SNPs are more significantly associated POAG in women than in men, suggesting the likely involvement of interactive effects among caveolins, eNOS and sex hormones^9^,^10^. Furthermore, we did not find gender preference in the association of rs1052990 with POAG, which could be due to the insufficient statistical power with the current sample size, confirmation of which requires further replication studies.

The SNP rs4236601 is located upstream to *CAV1*, while rs3801994 resides in the intronic region of *CAV1*. It has been shown that rs4236601 was not correlated with the expression of *CAV1* and *CAV2* in adipose tissue^7^ and in the retinas from normal donor eyes^8^. A recent study suggested an association between the allele G of rs17588172, a proxy SNP for rs1052990^11^, with decreased *CAV1* and *CAV2* mRNA in skin and adipose^15^. In this study, the SNP rs7801950, which is a proxy SNP for rs1052990, did not show a significant association with POAG (P = 0.22).

Biologically, the involvement of caveolins in different biological pathways suggests their possible effects in the eye. In adult mouse brain, genetic ablation of caveolin-1, −2 or −3 increased neural stem cell (NSC) proliferation in the subventricular zone^16^. Adult NSC forms the three major cell lineages of the central nervous system^16^,^17^,^18^. In the mouse, cerebral ischemia may induce increases in endothelial caveolin-1 and -2 protein levels. Cerebral ischemia in the *Cav-1* knockout mice resulted in an increased cerebral volume of infarction while in *Cav-2* knockout mice reduced infarction volume^19^. These findings supported the involvement of caveolin proteins in the ischemia process. Of note, a marked pathological change in glaucoma is the progressive loss of RGCs along with supporting glia and vasculature^1^. Ischemia plays a central role in apoptotic RGC death, and glaucoma patients can suffer from inadequate ocular blood flow^20^,^21^.

There were several limitations in this study. First, each of the study cohorts was of medium size, which may have limited the statistical power in identifying certain SNPs of mild effects, such as rs3801994, which showed a consistent trend of effect among the 3 Chinese cohorts but the P values in the Shantou and Beijing cohorts were not significant. The meta-analysis showed a marginally significant association between rs3801994 and POAG, with minimal inter-cohort heterogeneity (*I*^*2*^ = 0). Therefore, this association should be validated in more study cohorts. Second, only patients with high-tension glaucoma were enrolled in this study. Further studies are warranted to investigate the associations of the *CAV1*/*CAV2* SNPs with normal-tension glaucoma, which will further enrich our understanding of the roles of the *CAV1*/*CAV2* locus in POAG.

In summary, we confirmed the association of rs4236601 with POAG in the southern and northern Chinese, and identified a common SNP at the *CAV1*/*CAV2* locus, rs3801994, as a putative genetic biomarker for POAG in Chinese and Japanese, with diverse effects. Our findings enrich the understanding of the *CAV1*/*CAV2* locus in POAG, and provide new evidence on the ethnic diversities and allelic complexities in the genetic architecture of the disease.

## Methods

### Study subjects

This study included 4 cohorts from Hong Kong, Shantou (southern Chinese), Beijing (northern Chinese), and Osaka (Japanese). Part of the Chinese subjects from Hong Kong, Shantou and Beijing in the present study had been tested in our previous study^22^. All study subjects were unrelated. We recruited the Hong Kong subjects from the Hong Kong Eye Hospital and the Prince of Wales Hospital, Hong Kong. We recruited the Shantou subjects at the Joint Shantou International Eye Center of Shantou University and the Chinese University of Hong Kong, Shantou, China. The previous GWAS used part of the Hong Kong and Shantou subjects^7^. We recruited the Beijing cohort at the Beijing Tongren Eye Center, Beijing, China. We enrolled the Osaka cohort at the Department of Ophthalmology, Osaka University Hospital, Japan. We conducted this study in accordance with the tenets of the Declaration of Helsinki, under the ethics approvals from the ethics committees at respective collaborating institutions, such as the Chinese University of Hong Kong, the Joint Shantou International Eye Center of Shantou University, the Beijing Tongren Eye Center, and Osaka University Hospital. We obtained written informed consent from each subject prior to the study.

All study subjects received complete ophthalmic examinations. The diagnostic criteria for POAG at all recruiting centers were: (1) evident glaucomatous retinal nerve fiber layer loss, characteristic optic disc changes (i.e. optic nerve head notching, hemorrhage, and/or increased vertical cup/disc ratio), and typical visual field loss by standard automated perimetry with the Glaucoma Hemifield Test (Humphrey Field Analyzer; Carl Zeiss Meditec, Inc., Dublin, CA); (2) normal gonioscopic appearance with Shaffer grade III or IV open iridocorneal angle; (3) exclusion of secondary causes, e.g., trauma, uveitis, steroid effects, or exfoliation glaucoma; and (4) the highest IOP measured by using Goldmann applanation tonometry, was greater than 22 mmHg. We enrolled Chinese control subjects with age more than 60 years from the 3 recruiting centers in China, whilst we recruited the Japanese control subjects from the staff, volunteers and patients with mild cataract or mild refractive errors from the recruiting center in Osaka. All control participants received complete ophthalmic examination and were free of glaucoma or other major eye diseases, except for mild cataract and/or mild refractive errors if any. They had IOPs lower than 21 mmHg and open iridocorneal angle.

### SNP selection and genotyping

We investigated 8 SNPs in this study, including the SNP rs4236601 and rs1052990 that were reported in the previous GWAS^7^ and 6 haplotype-tagging SNPs covering the *CAV1*/*CAV2* locus based on the Chinese Han Beijing population in HapMap project (namely rs6466579, rs7801950, rs3779512, rs3807989, rs3801994 and rs1049337) with a r^2^ cut-off of 0.8 and MAF cut-off of 0.1 (Supplementary Table 4).

We extracted genomic DNA from whole blood using the QIAamp Blood Kit (QIAGEN, Hilden, Germany). We conducted genotyping using TaqMan SNP genotyping assays (Applied Biosystems, Pleasanton, CA, USA) on a Roche LightCycler 480 Real-Time PCR System (Roche Diagnostics; Basel, Switzerland) according to the manufacturer’s instructions. We genotyped all of the 8 SNPs in the Hong Kong subjects, and then genotyped 2 SNPs (rs4236601 and rs3801994), which showed an association (P < 0.05) with POAG in the Hong Kong cohort, in the Shantou, Beijing and Osaka subjects. Rs1052990 was also genotyped in the Osaka cohort as it was reported in GWAS^7^ and has been shown to be significant in a replication study in a Japanese population^12^.

### Statistical analysis

We compared mean age and gender proportions between the case and control groups using the student t-test and χ^2^ test, respectively. We assessed the HWE for individual SNP in the control groups using the χ^2^ test in PLINK (version 1.07; http://pngu.mgh.harvard.edu/~purcell/plink/)^23^, with a P < 0.05 indicating deviation from HWE. We compared the differences in the MAFs of the SNPs between cases and controls using χ^2^ test or Fisher’s exact test using PLINK. We used logistic regression analysis to adjust the association for age and gender, and condition on other SNPs to assess if an association was independent. We conducted subgroup analysis by gender to assess for gender-specific association. We performed the Breslow-day test for homogeneity of the ORs with the Tarone adjustment to determine if the ORs were significantly different between males and females^24^. We estimated population attributable risk of a SNP using the following equation, PAR% = 100 × p × (odds ratio−1) ÷ [p × (odds ratio−1) + 1], where p is the frequency of the associated alleles among control subjects^25^.

In association analysis, a P value of <0.05 suggested a potential association. To correct for multiple testing we used the SNPSpD program (Single Nucleotide Polymorphism Spectral Decomposition; http://gump.qimr.edu.au/general/daleN/SNPSpD/; accessed on 1 April, 2015)^26^. The SNPSpD takes into account inter-SNP correlations and generates the experiment-wide significance threshold that is required to keep the type I error rate of 5%. In this study, the effective number of independent markers was estimated to be 5, thus the significance threshold was 0.01 (0.05/5).

We pooled the data from the 3 Chinese cohorts by meta-analysis using the Review Manager (version 5.2.8; Copenhagen, The Nordic Cochrane Centre, The Cochrane Collaboration). We calculated the pooled-OR and 95% CI for each SNP using the fixed- or random-effect model based on inter-cohort heterogeneity. We assessed the heterogeneity by using the Q-statistic and the I^2 ^27. An I^2^ value of <24%, 25% to 49%, 50% to 74%, and ≥75% denoted none, low, moderate and high heterogeneity, respectively^27^,^28^. In this study, since the P value for the Q-statistic was >0.1 and the I^2^ < 50%, a fixed-effect model was adopted^29^.

## Additional Information

**How to cite this article**: Rong, S. S. *et al*. Ethnic specific association of the *CAV1/CAV2* locus with primary open-angle glaucoma. *Sci. Rep.* **6**, 27837; doi: 10.1038/srep27837 (2016).

## Supplementary Material

Supplementary Information

## Figures and Tables

**Table 1:** Demographic features of the study subjects.

Phenotype	Sample size	Age (year)	Gender (Male%)
Chinese - Hong Kong			
POAG	454	62 ± 15^a^	61.2%^a^
Control	436	74 ± 8	42.1%
Chinese – Shantou			
POAG	123	50 ± 21^a^	75.6%^a^
Control	731	72 ± 8	50.0%
Chinese – Beijing			
POAG	170	39 ± 16^a^	79.4%^a^
Control	200	69 ± 6	50.0%
Japanese – Osaka			
POAG	101	66 ± 11^a^	54.5%^a^
Control	207	43 ± 16	32.5%

POAG: primary open-angle glaucoma.

^a^Significantly different from control group (P < 0.05).

**Table 2:** Allelic association of the *CAV1/CAV2* SNPs with POAG.

SNP	Predicted function	MA	MAF		POAG vs. control		POAG vs. control^a^	
			Control	POAG	P	OR (95% CI)	P	OR (95% CI)
Chinese-Hong Kong			**n = 436**	**n = 454**				
rs1052990	c.*2195T > G	G	0.12	0.15	0.054	1.32 (0.99–1.75)	0.065	1.33 (0.98–1.79)
rs6466579	Intergenic	T	0.18	0.19	0.52	1.08 (0.85–1.37)	0.46	1.10 (0.85–1.42)
rs7801950	Intergenic	T	0.12	0.14	0.22	1.19 (0.90–1.58)	0.21	1.21 (0.90–1.62)
rs4236601	Intergenic	A	0.0043	0.02	**0.0072**	4.72 (1.36–16.36)	**0.0086**	6.25 (1.60–24.50)
rs3779512	c.102 + 4320T > G	T	0.061	0.056	0.68	0.92 (0.62–1.37)	0.93	0.98 (0.65–1.49)
rs3807989	c.103-12759A > G	A	0.22	0.25	0.083	1.21 (0.97–1.51)	0.11	1.21 (0.96–1.53)
rs3801994	c.103-8531G > A	A	0.15	0.18	**0.036**	1.31 (1.02–1.69)	**0.042**	1.32 (1.01–1.74)
rs1049337	c.*1246C > T	C	0.46	0.49	0.21	1.13 (0.93–1.36)	0.091	1.19 (0.97–1.46)
Chinese – Shantou			**n = 731**	**n = 123**				
rs4236601	Intergenic	A	0.0049	0.02	**0.0079**	4.23 (1.33–13.43)	**0.0037**	6.09 (1.80–20.63)
rs3801994	c.103-8531G > A	A	0.19	0.21	0.44	1.14 (0.82–1.59)	0.54	1.11 (0.80–1.54)
Chinese – Beijing			**n = 192**	**n = 170**				
rs4236601	Intergenic	A	0.013	0.036	0.057	2.86 (0.92–8.85)	**0.030**	3.92 (0.71–21.49)
rs3801994	c.103-8531G > A	A	0.19	0.2	0.67	1.09 (0.72–1.66)	0.85	1.04 (0.49–2.20)
Japanese – Osaka			**n = 207**	**n = 101**				
rs1052990	c.*2195T > G	G	0.25	0.19	0.11	0.71 (0.47–1.08)	**0.028**	0.55 (0.32–0.94)
rs4236601	Intergenic	–	–	–	–	–	–	–
rs3801994	c.103-8531G > A	A	0.33	0.28	0.22	0.79 (0.55–1.15)	**0.033**	0.58 (0.35–0.96)

*CAV1*: caveolin 1; *CAV2*: caveolin 2; CI: confidence interval; OR: odds ratio; MA: minor allele; MAF: minor allele frequency; POAG: primary open-angle glaucoma; SNP: single-nucleotide polymorphism.

^a^Outcomes adjusted for age and gender.

**Table 3:** Allelic association of the *CAV1/CAV2* SNPs with POAG in males and females.

SNP	MA	Male				Female				Breslow-day test^a^ (P)
		MAF Control	MAF POAG	P	OR (95% CI)	MAF Control	MAF POAG	P	OR (95% CI)	
Chinese - Hong Kong		**n** **=** **168**	**n** **=** **278**			**n** **=** **268**	**n** **=** **176**			
rs1052990	G	0.12	0.16	0.16	1.34 (0.89–2.02)	0.12	0.14	0.30	1.24 (0.82–1.88)	0.85
rs6466579	T	0.19	0.21	0.51	1.12 (0.80–1.58)	0.18	0.17	0.86	0.97 (0.68–1.38)	0.70
rs7801950	T	0.12	0.14	0.28	1.25 (0.83–1.88)	0.12	0.13	0.66	1.10 (0.73–1.65)	0.69
rs4236601	A	0.0034	0.017	0.083	5.21 (0.65–41.87)	0.005	0.024	**0.028**	4.94 (1.02–23.94)	0.65
rs3779512	T	0.072	0.063	0.61	0.87 (0.51–1.49)	0.054	0.045	0.58	0.84 (0.45–1.57)	0.95
rs3807989	A	0.22	0.27	0.078	1.33 (0.97–1.84)	0.22	0.23	0.8	1.04 (0.76–1.44)	0.45
rs3801994	A	0.15	0.19	0.079	1.39 (0.96–2.02)	0.15	0.17	0.36	1.19 (0.82–1.72)	0.67
rs1049337	C	0.46	0.48	0.61	1.07 (0.82–1.41)	0.46	0.51	0.18	1.20 (0.92–1.58)	0.67
Chinese - Shantou		**n** **=** **365**	**n** **=** **93**			**n** **=** **361**	**n** **=** **30**			
rs4236601	A	0.0014	0.016	**0.0071**	11.72 (1.21–113.30)	0.0084	0.033	0.067	4.06 (0.80–20.55)	0.70
rs3801994	A	0.19	0.23	0.33	1.21 (0.82–1.79)	0.19	0.17	0.67	0.86 (0.43–1.74)	0.56
Chinese - Beijing		**n** **=** **96**	**n** **=** **135**			**n** **=** **96**	**n** **=** **35**			
rs4236601	A	0.016	0.019	0.81	1.19 (0.28–5.03)	0.01	0.057	**0.025**	5.76 (1.03–32.16)	0.56
rs3801994	A	0.2	0.23	0.46	1.19 (0.75–1.87)	0.18	0.15	0.57	0.80 (0.37–1.73)	0.49
Japanese - Osaka		**n** **=** **68**	**n** **=** **55**			**n** **=** **139**	**n** **=** **46**			
rs1052990	G	0.27	0.19	0.14	0.63 (0.34–1.16)	0.23	0.18	0.34	0.75 (0.41–1.36)	0.78
rs4236601	A	–	–	–	–	–	–	–	–	–
rs3801994	A	0.38	0.31	0.23	0.72 (0.42–1.23)	0.3	0.24	0.24	0.72 (0.42–1.24)	0.99

*CAV1*: caveolin 1; *CAV2*: caveolin 2; CI: confidence interval; MA: minor allele; MAF: minor allele frequency; OR: odds ratio; POAG: primary open-angle glaucoma; SNP: single-nucleotide polymorphism.

^a^Breslow-day test was performed using Taron adjustment.
