# Supplementary material for: Ethnic specific association of the CAV1/CAV2 locus with primary open-angle glaucoma
Source: Sci Rep. 2016 Jun 14;6:27837. doi: 10.1038/srep27837 (PMC4906515; doi:10.1038/srep27837)
Supplement: Supplementary Information [file srep27837-s1.pdf]

Ethnic specific association of the *CAV1/CAV2* locus with primary open-angle glaucoma

Shi Song Rong[1*], Li Jia Chen[1,2*], Christopher K.S. Leung[1*], Kenji Matsushita[3*], Liyun Jia[4], Atsuya Miki[3], Sylvia W.Y. Chiang[1], Pancy O.S. Tam[1], Noriyasu Hashida[3], Alvin L. Young[2], Motokazu Tsujikawa[3], Mingzhi Zhang[5], Ningli Wang[4], Kohji Nishida[3], Chi Pui Pang[1,2,5]

* These authors contributed equally to this study.

[1]Department of Ophthalmology and Visual Sciences, the Chinese University of Hong Kong, Hong Kong, China;

[2]Department of Ophthalmology and Visual Sciences, Prince of Wales Hospital, Hong Kong, China;

[3]Department of Ophthalmology, Osaka University Graduate School of Medicine, Suita, Osaka, Japan;

[4]Beijing Tongren Eye Center, Beijing Tongren Hospital, Capital Medical University, Beijing, China;

[5]Joint Shantou International Eye Center of Shantou University and the Chinese University of Hong Kong, Shantou, China.

Number of supplementary information: 4 supplementary tables and 1 supplementary figure

Correspondence:

Dr. Li Jia Chen

Department of Ophthalmology and Visual Sciences, the Chinese University of Hong Kong

Hong Kong Eye Hospital, 147K, Argyle Street, Kowloon, Hong Kong.

Phone: +852 39435810 Fax: +852 27159490 E-mail: lijia_chen@cuhk.edu.hk

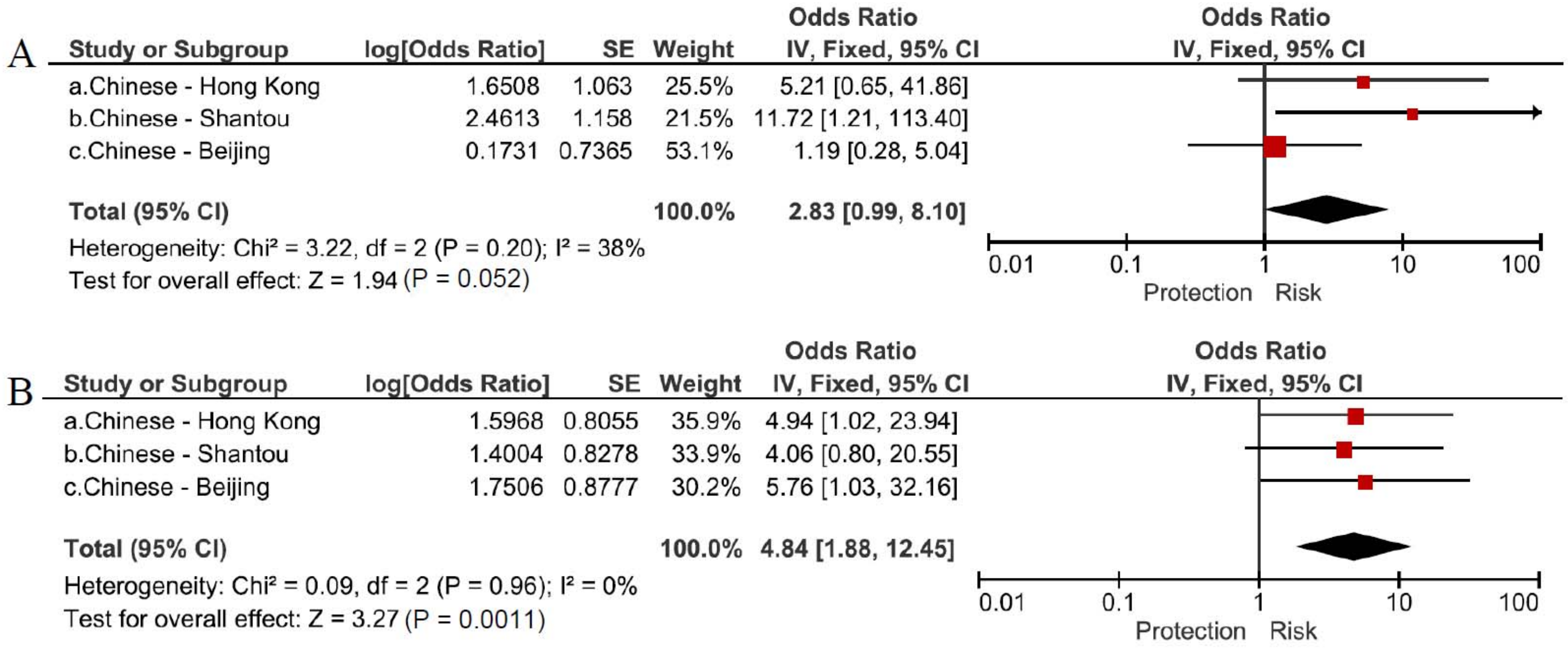

Supplementary Fig S1. Association of rs4236601 with primary open-angle glaucoma in male and female subjects

A. Meta-analysis performed in Chinese male subjects; B. Meta-analysis performed in Chinese female subjects

Supplementary Table S1. Quality control results

No.	SNP	Chr: position [a]	Genotype call rate in all samples	Test for HWE in controls (P)
**Chinese - Hong Kong**			**n=881**	
1	rs1052990	7: 116148370	0.980	0.35
2	rs6466579	7: 116151155	0.994	0.87
3	rs7801950	7: 116154783	0.995	0.17
4	rs4236601	7: 116162729	0.988	1.00
5	rs3779512	7: 116171063	0.992	0.061
6	rs3807989	7: 116186241	0.995	0.78
7	rs3801994	7: 116190469	0.994	0.45
8	rs1049337	7: 116200587	0.989	1.00
**Chinese – Shantou**			**n=854**	
1	rs4236601	7: 116162729	0.984	1.00
2	rs3801994	7: 116190469	0.999	0.19
**Chinese – Beijing**			**n=370**	
1	rs4236601	7: 116162729	1.00	1.00
2	rs3801994	7: 116190469	1.00	0.64
**Japanese – Osaka**			**n=308**	
1	rs1052990	7: 116148370	0.996	0.85
2	rs4236601	7: 116162729	1.00	Monomorphic
3	rs3801994	7: 116190469	1.00	0.87

[a] The position was aligned to GRCh37/hg19.

Chr: chromosome; HWE: Hardy-Weinberg equilibrium; SNP: single-nucleotide polymorphism.

Supplementary Table S2. Association of rs4236601 with primary open-angle glaucoma conditioned on the other single nucleotide polymorphisms

No.	Adjusted SNP	MA	POAG vs. controls	
			P	OR (95% CI)
1	rs1052990	G	**0.021**	4.43 (1.25-15.66)
2	rs6466579	T	**0.014**	4.80 (1.38-16.76)
3	rs7801950	T	**0.010**	5.13 (1.47-17.86)
4	rs4236601	A	n.a.	n.a.
5	rs3779512	T	**0.0080**	5.62 (1.57-20.16)
6	rs3807989	A	**0.016**	4.62 (1.33-16.13)
7	rs3801994	A	**0.0088**	5.31 (1.52-18.50)
8	rs1049337	C	**0.011**	5.08 (1.46-17.72)

CI: confidence interval; MA: minor allele; n.a.: not applicable; OR: odds ratio; POAG: primary open-angle glaucoma; SNP: single-nucleotide polymorphism

Supplementary Table S3. Association of rs3809916 with primary open-angle glaucoma conditioned on the other single nucleotide polymorphisms

No.	Adjusted SNP	MA	POAG vs. controls	
			P	OR (95% CI)
1	rs1052990	G	0.10	1.31 (0.95-1.79)
2	rs6466579	T	**0.035**	1.36 (1.02-1.80)
3	rs7801950	T	0.091	1.31 (0.96-1.80)
4	rs4236601	A	**0.038**	1.22 (1.00-1.61)
5	rs3779512	T	0.055	1.29 (0.99-1.67)
6	rs3807989	A	0.159	1.32 (0.90-1.94)
7	rs3801994	A	n.a.	n.a.
8	rs1049337	C	**0.038**	1.31 (1.02-1.70)

CI: confidence interval; MA: minor allele; n.a.: not applicable; OR: odds ratio; POAG: primary open-angle glaucoma; SNP: single-nucleotide polymorphism

Supplementary Table S4. Selection of SNPs to be tested in the *CAV1/CAV2* locus

No.	SNP	NCBI36/hg18	Distance to a gene (bp)	MA	MAF in CHB	Reason for selection	Reference
1	rs1052990	c.*2195T>G, downstream of *CAV2*	2,677	G	0.173	POAG GWAS	1
2	rs6466579	g.115938641T>C, downstream of *CAV2*	2,810	T	0.278	Tag SNP	-
3	rs7801950	g.115942269C>T, downstream of *CAV2*	6,438	T	0.189	Tag SNP	-
4	rs4236601	g.115949965G>A, upstream of *CAV1*	2,110	A	0.017	POAG GWAS	1
5	rs3779512	*CAV1* c.102+4320T>G	Intron 2	T	0.100	Tag SNP	-
6	rs3807989	*CAV1* c.103-12759A>G	Intron 2	A	0.289	Tag SNP; ECG P-R interval & atrial fibrillation	2
7	rs3801994	*CAV1* c.103-8531G>A	Intron 2	A	0.189	Tag SNP	-
8	rs1049337	*CAV1* c.*1246C>T	1,246	T	0.477	Tag SNP	-

CAV1: caveolin 1; *CAV2*: caveolin 2; CHB: Chinese Han Beijing; ECG: electrocardiogram; MA: minor allele; MAF: minor allele frequency; NCBI: National Center for Biotechnology Information; SNP: single nucleotide polymorphism; GWAS: genome-wide association study

Reference

1 Thorleifsson, G. *et al.* Common variants near CAV1 and CAV2 are associated with primary open-angle glaucoma. *Nat Genet* **42**, 906-909, (2010).

2 Holm, H. *et al.* Several common variants modulate heart rate, PR interval and QRS duration. *Nat Genet* **42**, 117-122, (2010).
